# Robot-assisted technique can achieve accurate screw placement in four-corner fusion and reduce operative difficulty: a cadaver study

**DOI:** 10.1186/s13018-024-05213-w

**Published:** 2024-11-06

**Authors:** Zhixin Wang, Bo Liu, Zhe Yi, Ke Xu, Shijie Jia, Qianqian Wang, Yaobin Yin

**Affiliations:** 1 Department of Hand Surgery, Beijing Jishuitan Hospital, Capital Medical University, No.31, Xin Jie Kou East Street, Xi Cheng District, Beijing, 100035 China; 2 Beijing Research Institute of Orthopedics, No. 31, Xin Jie Kou East Street, Xi Cheng District, Beijing, 100035 China

**Keywords:** Four-corner fusion, Robotic surgical procedures, Four-corner arthrodesis, Robot-assisted technique, Orthopaedic robot

## Abstract

**Background:**

The purpose of this study is to explore the feasibility and accuracy of a robot-assisted technique in four-corner fusion compared with traditional freehand operation.

**Methods:**

Twenty cadaver specimens were randomly assigned to the robot-assisted group and freehand groups. Three screws were placed percutaneously to fix the capitate-lunate joint, lunate-triquetrum joint, and triquetrum-hamate-capitate joint in each specimen by robot-assisted or freehand technique. The offset between the actual and planned screw positions was determined by merging the images of intraoperative and postoperative CT scans in the robot-assisted group. The centrality of the screw, time-consuming, drilling attempts, and radiation exposure were compared between the two groups.

**Results:**

The mean offset between the actual and planned screw position was 1.09 (SD: 0.56) mm. The offset at the start point of the screw was significantly lower than that at the endpoint. There was no significant difference in the centrality of the screws, surgical time between the two groups. The number of drilling attempts and the radiation dose received by surgeons were significantly lower in the robot-assisted group.

**Conclusions:**

Although there was no significant difference in screw centrality between the two groups, the slight offset between the actual and planned screw positions confirmed the feasibility of the robot-assisted technique in four-corner fusion. The robot-assisted technique has advantages in reducing the difficulty of surgery and protecting the surgeon from exposure to large doses of radiation.

## Background

Four-corner fusion (4CF), proposed by Watson in 1984, is a commonly used limited fusion technique for treating osteoarthritis caused by scaphoid nonunion advanced collapse (SNAC) and scapholunate advanced collapse (SLAC) [1, 2]. This procedure involves scaphoid ectomy and fusion of the capitate, lunate, triquetrum, and hamate [3]. While effectively reducing pain, 4CF also preserves some range of wrist motion and grip strength [4].

The fixation methods of 4CF include pins, plates, and cannulated screws. Due to the compression effect and the low incidence of implant-related complications, cannulated screw fixation has been one of the most widespread methods in recent years [5–7]. However, 4CF using cannulated screw fixation is technically difficult in both open and arthroscopic surgery. Aleksi Vihanto’s research has shown that 4CF by percutaneous cannulated screw fixation is technically demanding and requires a long learning curve, with surgical time fluctuating between 2 and 4 h [8]. Ho’s study also showed that surgeons need to complete five operations to decrease surgery time [9, 10]. In addition, even if the operation is performed by an experienced surgeon, complications such as screw perforation and fixation failure can still occur [11].

Orthopaedic robots have been successfully applied to the wrist, and previous reports mainly focused on treating scaphoid fractures [12–14]. Compared with scaphoid fracture, the robotic technique may have a greater value in 4CF. It can not only rationally plan the position and length of multiple screws but also may improve the accuracy of screw placement and reduce the difficulty of surgery. There have been attempts to complete 4CF with robot assistance in clinical practice, but it is still limited to case reports [15]. This study intends to explore the feasibility of applying the robot-assisted technique to 4CF through cadaveric experiments and evaluate whether the robot-assisted technique has advantages in the accuracy and convenience of screw placement compared with the traditional freehand operation.

## Methods

This study was performed in accordance with the principles of the Declaration of Helsinki (revised in 2013). The ethics committee of our hospital reviewed and approved the experiment.

### Specimen

Ten pairs of forearm specimens were used in this experiment. All specimens were amputated above the elbow and appeared normal under inspection without skin surface collapse. Five left specimens and five right specimens were randomly selected into the robot-assisted group, and the other ten specimens were selected into the freehand group.

## Robot-assisted technique

**Fig. 1:**
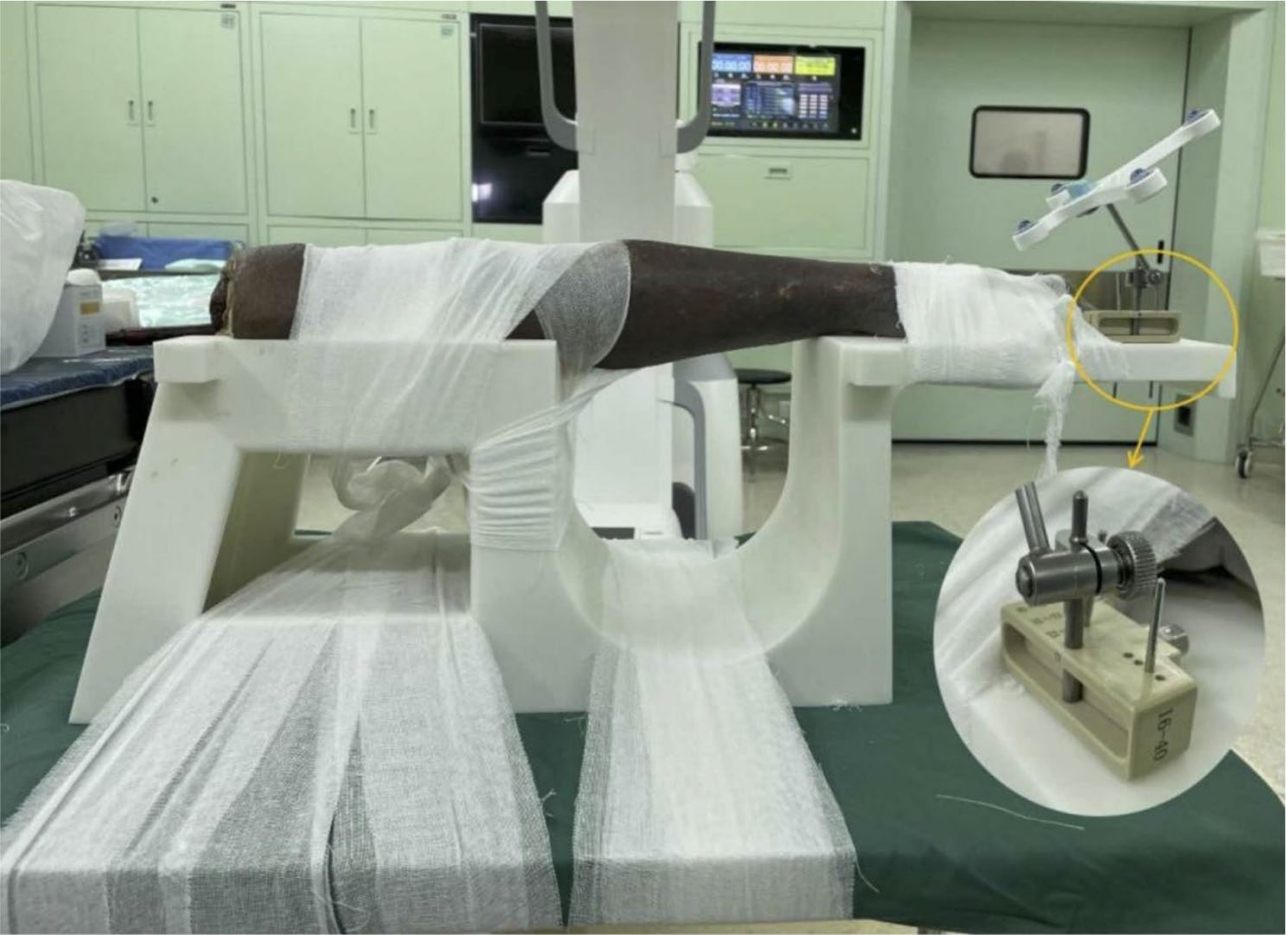
Fixation of specimens and connection of patient tracker in robot-assisted group. The partially enlarged image marked with a yellow circle shows the indirect rigid connection between the target bone and the patient tracker

**Fig. 2:**
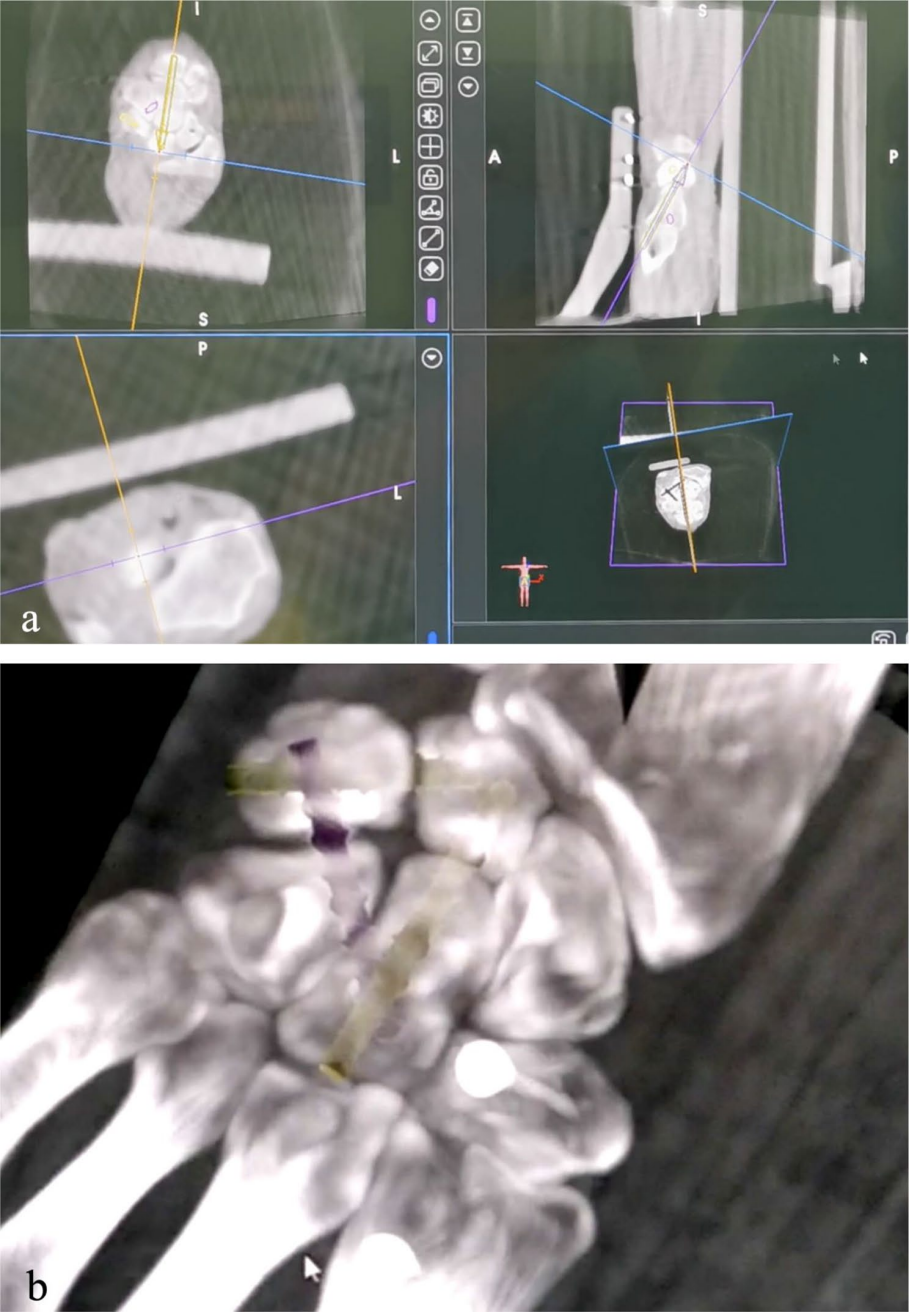
The surgeon planned the trajectory of the screws in the software workstation of TIRobot (**a**) and checked the planned position of three screws in 3D image (**b**). The entry point, end point and length of the screws are repeatedly adjusted so that the screws were centered as much as possible to hold more bone, while avoiding interference between screws

The forearm was fixed in pronation with a bandage on a customized X-ray transparent fixator, and the patient tracker was connected to the fixator (Fig. 1). Tianji Robot (TINAVI Medical Technologies Co., Ltd., Beijing, China) assisted the surgery. The system comprises a preoperative planning workstation, an optical tracking device, and a robotic arm. The 3D C-arm scanning system (ISO C3D, Siemens, Germany) collected the image data of the wrist joint and sent it to the workstation. The system automatically recognized the spatial data of the carpal bones according to the patient tracker. The surgeon planned the position and length of the screws on the 3D images. Then the robotic arm automatically moved to the designated position according to the plan. Three screws were planned in sequence. The first is the capitate-lunate screw (CL screw), starting at the dorsal edge of the distal capitate and ending at the middle portion of the lunate. The second is the lunate-triquetrum screw (LT screw), starting at the ulnar edge of the triquetrum and ending at the middle portion of the lunate (dorsal to the CL screw). The third is the triquetrum-hamate-capitate screw (THC screw), starting at the ulnar edge of the triquetrum (dorsal to the LT screw) and ending at the distal radial portion of the capitate. After determining the entry point and end point of the screw, the system simulated the screws with a diameter of 3.5 mm and adjusted the length. The position and length of the screws were repeatedly confirmed and adjusted in the coronal, sagittal, and axial views (Fig. 2a) to ensure that the screws were completely buried in the bone. The three screws did not interfere with each other (Fig. 2b). Then, the robotic arm carried a 1.1 mm cannula and automatically moved to the planned position. The guide pins were inserted manually according to the cannula. After the intraoperative fluoroscopy initially confirmed the correct position of the guide pins, the screws were placed in sequence. The wrist joint was then scanned again with the C-arm to obtain 3D reconstruction data for evaluating the actual position of the screws.

## Traditional freehand technique

Percutaneous cannulated screw fixation was used in the freehand group. The forearm was pronated and fixed on the table, and a guide pin was placed from the distal dorsal margin of the capitate to the proximal volar side to fix the capitate-lunate joint. The second guide pin was inserted from the ulnar side of the triquetrum to fix the lunate-triquetrum joint. The third guide pin was placed from the ulnar side of the triquetrum to fix the triquetrum and hamate to the distal radial portion of the capitate. The position of the guide pins was adjusted under fluoroscopy to hold as much bone as possible while leaving enough distance between each other to prevent screws from interfering with each other. Then the screws were inserted in sequence. C-arm scanning was used to obtain 3D images after screw placement.

## Data collection

**Fig. 3:**
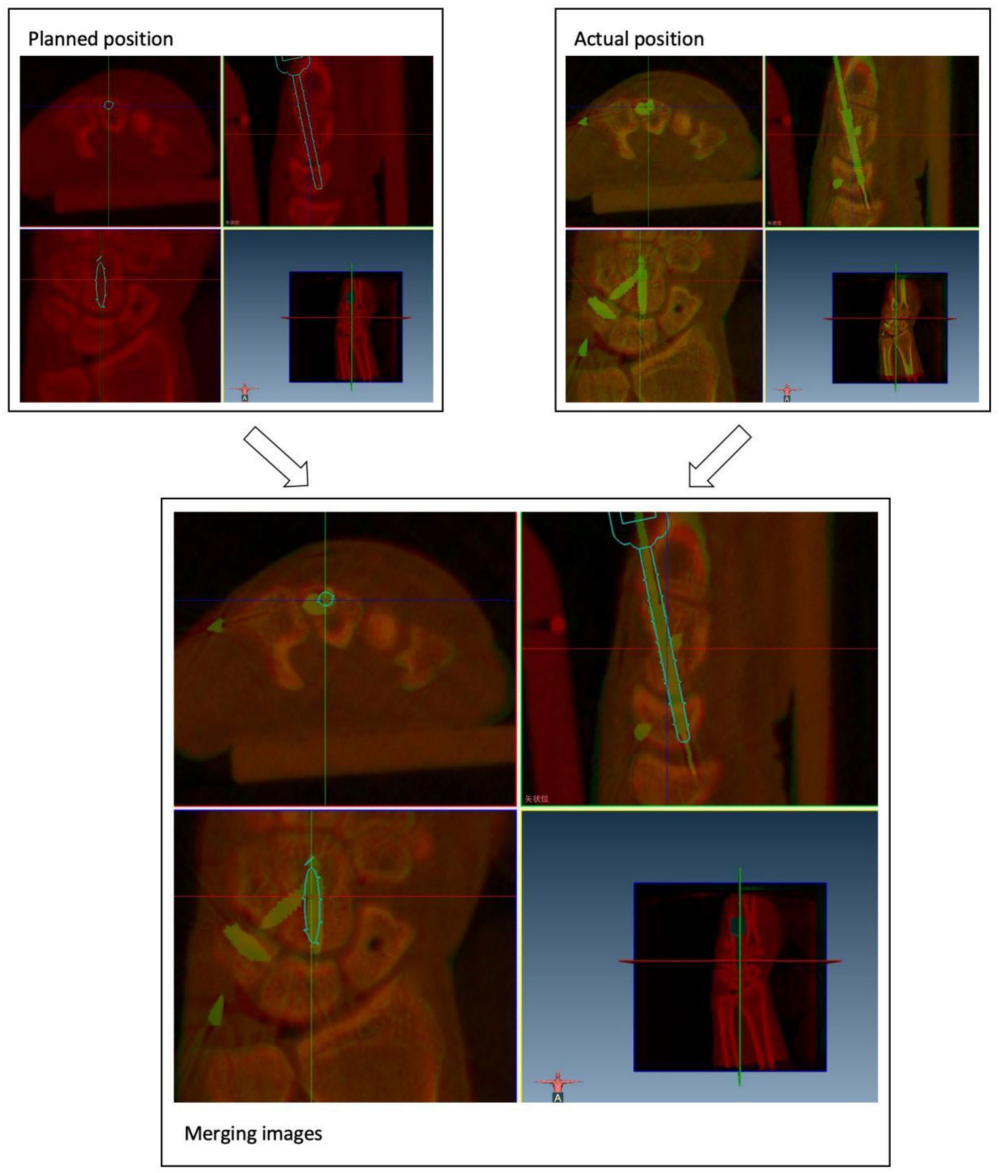
After alignment of the planned image and postoperative validation image, merging image was obtained, and the offset between the planned and actual position of screws was achieved through the coordinate information on the image

All operations in both groups were performed by the same two hand surgeons with more than five years of experience in wrist surgery and research (Level 4 of expertise according to Tang and Giddins [16]). The accuracy of the screw placement was assessed according to the offset between the planned trajectories and the final screw positions. The offset was measured by merging the postoperative computed tomography (CT) images with the preoperative planning images at both the screw entry point and its endpoint, then averaging these two deviations (Fig. 3). We also compared the screw offset at the entry and endpoints to assess the effect of intercarpal micromotion on screw placement accuracy.

**Fig. 4:**
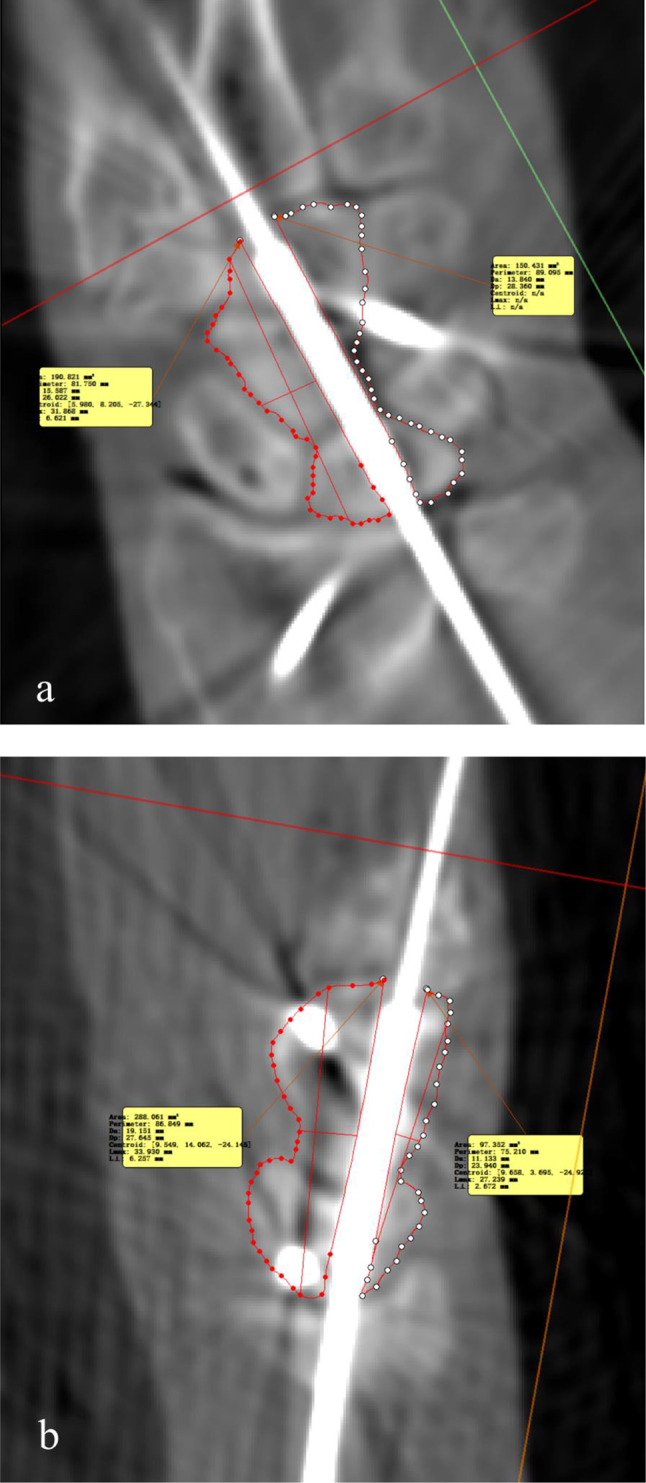
The postoperative images were reconstructed along the axis of the screw to reveal the whole length of the screw. The area of carpal bones on both sides of the screw was measured in two orthogonal planes (**a**: resliced coronal view; **b**: resliced sagittal view). The relative centrality of the screw was determined by the average of the area difference between the two sides of the screw. The smaller the average, the more centered the screw was

The centrality of screws in the robot-assisted group and the freehand group was compared. Because the screws spanned two or three carpal bones, there is no ideal central axis. Therefore, we evaluated the relative centrality of the screw by the area differences of carpal bones on both sides of the screw. The postoperative images were reconstructed at the screw plane to reveal the whole length of the screw and its position in carpal bones. In these multiplanar reconstructed (MPR) images, the area differences of carpal bones on both sides of the screw were measured in two orthogonal planes (Fig. 4). The smaller the mean of the area differences in the two planes, the more centered the screw was. The data of centrality analysis was measured separately by two surgeons, and the measurement was repeated two weeks later.

The other outcome measures include: (1) The number of drilling attempts of the guidewire and (2) the total operation duration were recorded by one participant during the execution of the procedure; (3) Total fluoroscopy times and radiation dose were recorded automatically by the fluoroscopic equipment and 3D C-arm device; (4) The incidence of screw interference and screw perforation through the cortex were assessed by the postoperative imaging.

### Statistical analysis

Continuous variables are presented as the means and standard deviations (SDs) or median (interquartile range). Normality was checked using the Shapiro-Wilk test. The specimens in the two groups were paired left and right (considered to be anatomically identical), therefore the paired t-test or the Wilcoxon Signed Rank test were used to compare the parametric or nonparametric data of the two groups, respectively. A generalized estimation equation was used to evaluate the differences between three or more correlated groups. The data analysis was conducted using SPSS software (SPSS 21.0). A significant difference was defined by an *α* level of 0.05 in two-sided. Intraclass correlation coefficient (ICC) was used to measure intra-observer and test-retest reliability. The agreement levels were classified as poor (ICC < 0.5), moderate (ICC 0.5–0.75), good (ICC 0.75–0.9), and excellent (ICC > 0.9).

## Results

The accuracy of robot-assisted screw placement was measured through merging images. The mean offset of all screws between the actual and planned position was 1.09 (SD: 0.56) mm. There was no significant difference in the offset between CL, LT, and THC screws. While in the comparison of the offset at the start and end point of the screws, we found that the offset at the start point was significantly lower than that at the endpoint (Table 1).

**Table 1:** The comparison of the offset between screws

	Mean (SD) offset (mm)	Sig. of Shapiro-Wilk test	*p* value
CL screw	0.9 (0.4)	0.965	0.191*
LT screw	1.2 (0.7)	0.507	
THC screw	1.2 (0.5)	0.737	
Start point of all screws	0.8 (0.5)	0.505	< 0.001#
End point of all screws	1.4 (0.8)	0.238	

* The *p* value was used to evaluate the offset differences between the three groups of CL, LT and THC screw

# The *p* value was used to evaluate the offset differences between the start point and end point

**Fig. 5:**
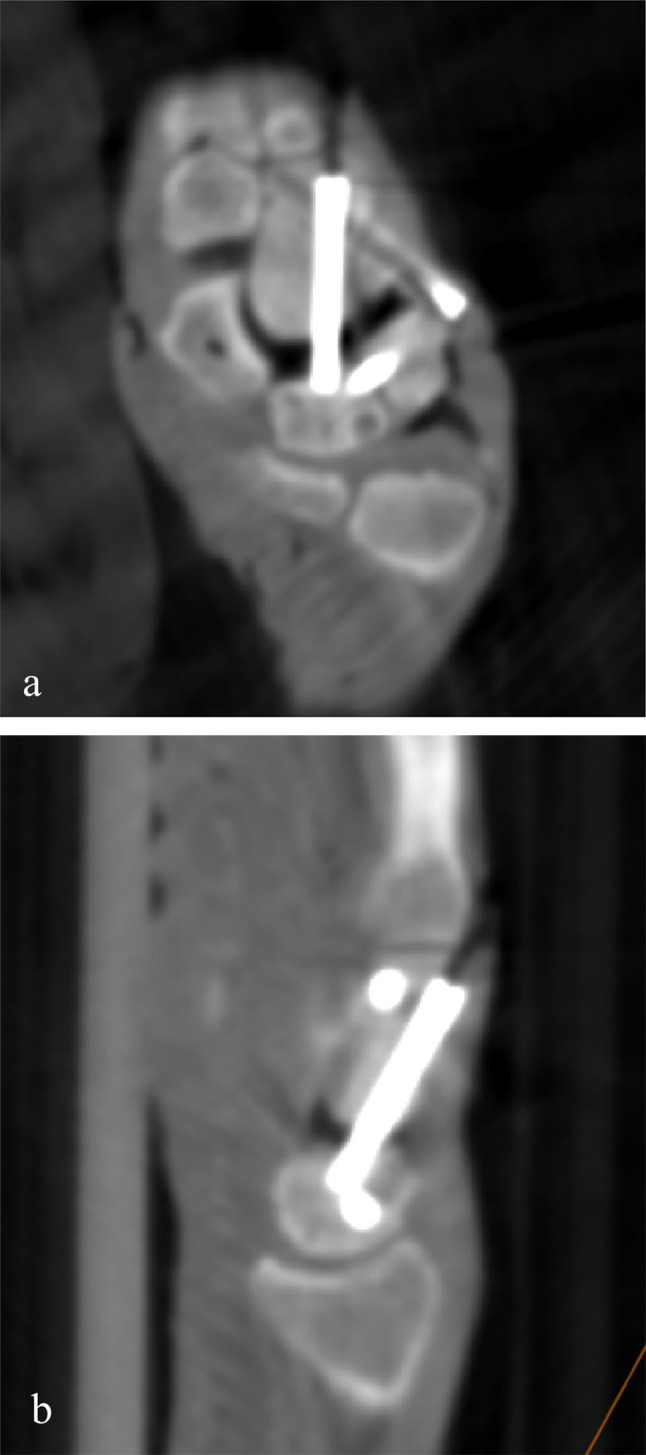
Illustration of “screw fighting” between the CL and LT screw and the resulting widening of the midcarpal joint space in the resliced coronal view (**a**) and resliced sagittal view (**b**)

There was no significant difference in the relative centrality of the screws between the two groups. Compared to the freehand group, the difference in bone area on both sides of the screw in the robot-assisted group was smaller. Still, the difference between the two groups was not statistically significant (Table 2). The ICC for inter-observer reliability in the measurement of screw centrality was 0.88 (95% CI: 0.63–0.96). No evident screw penetration of the cortex was found in either group. However, in the freehand group, 3 cases of “screw fighting” were found due to the proximity of screws (Fig. 5).

**Table 2:** Comparative data of the two groups

	Robot-assisted		Freehand		*p* value
	Median (interquartile range)	Sig. of Shapiro-Wilk test	Median (interquartile range)	Sig. of Shapiro-Wilk test	
Difference of bone area on both sides of the screw (mm^2^)	70.8 (48.8, 106.8)	0.869	85.3 (39.7, 126.9)	0.037	0.393
Operation time (min)	39.5 (32.8,46.5)	0.756	32.0 (26.0,49.0)	0.604	0.61
Drilling attempts	3.0 (3.0,3.8)	0.000	19.5 (6.5,36.3)	0.239	0.005
Fluoroscopy times	11.0 (8.0,23.3)	0.009	86.5 (56.0,118.0)	0.321	0.005
Total radiation dose (mGy)	7.3 (6.7,9.3)	0.008	1.7 (1.3,2.1)	0.865	0.005
Radiation dose received by surgeon (mGy)	0.5 (0.4,1.5)	0.002	1.7 (1.3,2.1)	0.865	0.047

Although the drilling attempts in the robot-assisted group were significantly lower than those in the freehand group, the overall surgical time was comparable between the two groups. The radiation dose borne by the surgeons in the robot-assisted group was significantly lower than that of the freehand group, due to the need for wrist CT data collection (Table 2).

## Discussion

The orthopedic robot has been preliminarily applied to the wrist joint. Liu et al. first reported the clinical outcomes of robot-assisted fixation of acute scaphoid fracture in 2019, which initially confirmed the feasibility of applying the robot technique to the wrist [17]. Subsequently, CW Xiao et al., through a retrospective study, compared the clinical outcomes of robot-assisted and freehand fixation in the treatment of scaphoid fracture. The results showed that the screw position under robot assistance was significantly better than that under freehand operation, and the former used less surgical time and fewer drilling attempts [13]. YB Yin et al., using cadaver specimens, compared the performance of robot-assisted and freehand fixation in scaphoid fracture, and the results showed that there was no significant difference in the accuracy of screw placement [14].

Compared with the fixation of scaphoid fracture, 4CF is technically more difficult for the surgeon. When multiple screws need to be inserted, the freehand operation requires repeated fluoroscopy and adjustment to ensure reliable screw fixation and no interference with each other. This requires the surgeon to clearly understand the three-dimensional structure of the carpal bones when placing the guide pins and planning the target path of each guide pin. The results of this experiment showed that there were 3 cases of “screw fighting” in the freehand group, which was difficult to be identified through intraoperative fluoroscopy.

In robot-assisted surgery, the surgeon can plan the ideal position of the screw at the coronal, sagittal, and axial views, which theoretically allows the screw to be relatively centered, thus holding more bone and making the fixation more reliable. In this study, the relative centrality of the screws was evaluated by the area difference of carpal bones on both sides of the screws, which is smaller in the robot-assisted group. However, the difference between the two groups was not statistically significant.

In previous studies of robot-assisted fixation of scaphoid fracture, the accuracy of screw placement was evaluated only by comparing the screw axis in postoperative CT images with the ideal axis determined subjectively by the surgeon without directly measuring whether the actual screw path was consistent with the planned path in robotic surgery [13, 14]. Unlike the treatment of the scaphoid fracture, 4CF requires fixation of 2 or 3 carpal bones. The intercarpal micromotion may adversely affect the accuracy of screw placement. Therefore, we measured deviations between the actual and planned screw positions at the start and end points by emerging the planned and postoperative CT images. The results showed that the mean offset at the start point of the screws was 0.82 (SD: 0.51) mm, which was similar to the performance of the robot-assisted technique in other sites of surgery [18]. However, the mean offset at the end point of the screws was 1.37 (SD:0.79) mm, which was significantly higher than the offset at the start points, suggesting that intercarpal micromotion does adversely affect the accuracy of screw placement. In practical clinical application, widening the intercarpal space after cartilage removal may increase the error caused by the micromotion between carpal bones.

In addition, there was no significant difference in surgical time between the robot-assisted and freehand groups. This is because the robot-assisted group spent of much time in the process of image data collection, equipment installation, and regulation. The number of drilling attempts in the robot-assisted group was significantly lower than in the freehand group. This parameter is the most intuitive index reflecting the difficulty of the operation, indicating that the robot technique can substantially reduce the difficulty of 4CF. More importantly, robot assistance may also reduce the potential risks of iatrogenic nerve and tendon injury caused by repeated drilling. At the same time, we compared the dose of radiation exposure during surgery between the two groups. The overall radiation dose of the robot-assisted group was higher than that of the freehand group because the 3D image data of the wrist needed to be collected during the operation. However, when it comes to the number of times the surgeon was exposed to fluoroscopy and the amount of radiation they received, the robot-assisted group was significantly lower than the freehand group. For orthopedic surgeons, reducing the risk of occupational exposure to intraoperative radiation is also one of the important advantages of the robot-assisted technique.

During the experiment, we also found problems in applying the robot-assisted technique. The way the patient tracker is connected and the wrist fixation device still needs to be improved. The rigid connection between the patient tracker and the target bone is a prerequisite for robot accuracy [18]. In this study, an indirect connection of the patient tracker to the target bone was achieved through a specially designed fixator that tightly immobilized the wrist joint. The cumbersome fixation steps and the cramped operating space during bone tunnel drilling still needed to be optimized to further improve the accuracy of robotic surgery and reduce surgical time.

There are some limitations in this study. Firstly, our experiment simulated percutaneous screw fixation, but due to the limited number of specimens, we did not compare the differences between robot-assisted and freehand screw placement in open surgery scenarios. Secondly, we did not perform procedures such as cartilage removal and cancellous bone grafting, which may increase intercarpal micromotion in real surgery. Therefore, our results may overestimate the accuracy of robot-assisted screw placement to some extent.

## Conclusions

Overall, our results indicate that robot-assisted technique can be applied to more complex wrist joint surgeries in addition to scaphoid fractures. Although there was no significant difference in screw centrality between the two groups, the slight offset between the actual and planned screw positions confirmed the feasibility of robot-assisted technique in four-corner fusion. The robot-assisted technique has advantages in reducing the difficulty of surgery and protecting the surgeon from exposure to large doses of radiation.

## Data Availability

No datasets were generated or analysed during the current study.
